# Novel plasmid-mediated colistin resistance *mcr-4 g*ene in *Salmonella* and *Escherichia coli*, Italy 2013, Spain and Belgium, 2015 to 2016

**DOI:** 10.2807/1560-7917.ES.2017.22.31.30589

**Published:** 2017-08-03

**Authors:** Alessandra Carattoli, Laura Villa, Claudia Feudi, Ludovica Curcio, Serenella Orsini, Andrea Luppi, Giovanni Pezzotti, Chiara Francesca Magistrali

**Affiliations:** 1 Department of Infectious Diseases, Istituto Superiore di Sanità, Rome, Italy; 2 Institute of Microbiology and Epizootics, Centre for Infection Medicine, Department of Veterinary Medicine, Freie Universität Berlin, Berlin, Germany; 3 Istituto Zooprofilattico Sperimentale dell'Umbria e delle Marche, Perugia, Italy; 4 Istituto Zooprofilattico Sperimentale della Lombardia e dell’Emilia Romagna, Reggio Emilia, Italy

**Keywords:** colistin, ColE, Salmonella, mcr

## Abstract

A novel *mcr* colistin resistance gene was identified in a strain of *Salmonella enterica*, monophasic variant of serovar Typhimurium (4,5,12:i:- ), isolated from a pig at slaughter in Italy in 2013, and in *Escherichia coli* strains collected during routine diagnostic of post-weaning diarrhoea in pigs from Spain and Belgium in 2015 and 2016. Immediate implementation of *mcr*-screening including this novel gene variant is required for *Salmonella* and *E. coli* from humans and food-producing animals in Europe.

The wide use of colistin in veterinary medicine for the control of *Enterobacteriaceae* infections, and for prophylaxis purposes caused a significant increase in colistin resistance, especially in *Escherichia coli* from pigs [1]. 

The primary objective of this study was the identification of an unknown mechanism of colistin resistance in a *Salmonellaenterica* serovar Typhimurium (4,5,12:i:- ), of our collection [2]. The relevance of the new *mcr-4* gene, for the first time here identified, was supported by its occurrence in numerous *Escherichia coli* of swine origin recently isolated from different European countries. 

## A novel *mcr-*gene in *Salmonella* with origins from *Shewanella frigidimarina*

In 2013, one colistin-resistant (minimum inhibitory concentration (MIC) = 8 mg/L, measured by broth microdilution) [3] monophasic variant of *S. enterica* serovar Typhimurium (4,5,12:i:– ) isolate R3445 was obtained from the caecal content of a pig at slaughter, during a study performed by the Istituto Zooprofilattico of Umbria and Marche in Italy [2]. The pig originated from a small finishing herd in central Italy [2].

Whole genome sequencing (WGS) of the strain was performed by an Illumina MiSeq (2 x 300 PE), with the objective to identify the mechanism of colistin resistance. The R3445 genome was negative for *mcr-1, mcr-2* and *mcr-3* genes. Genome comparative analysis, performed against the *S. enterica* Typhimurium LT2 genome (NC_003197.2) identified eight contigs > 1 kb that were not present in the reference genome. Three of them contained the chromosomally located resistance genes *bla*_TEM-1B_, *strA-strB-sul2* and *tetB*, respectively (identified by ResFinder https://cge.cbs.dtu.dk/services/) [4]. In the remaining five divergent regions, PlasmidFinder (https://cge.cbs.dtu.dk/services/) detected ColE-like replicons [5]. Assembly and annotation analysis suggested the presence of five small extrachromosomal plasmids in the colistin-resistant R3445 *Salmonella* strain; large plasmids were not identified (Table 1).

**Table 1:** Characteristics of the *mcr*-*4* positive strains analysed in this study, Italy 2013, Spain and Belgium, 2015 to 2016 (n = 4), and their transformants and conjugants

Strain	Country	MLST	Plasmid content	Plasmid-mediated colistin resistance	Additional resistance genes	Colistin MIC mg/L
**R3445** ***Salmonella enterica***	Italy	34	ColE10, ColRNAI_34, ColRNAI_36, ColRNAI_38, ColRNAI_46	*mcr-4*	*bla*_TEM-1B_, *strA, strB, sul2, tet(B)*	8
**3445T (transformant in DH5-α**)			ColE10	*mcr-4*		2
**DH5-α **						0.25
**R4287** ***Escherichia coli***	Spain	10 (CC10)	I1, I2, FII, FIB, FIC, HI2, Col156, ColRNAI_34, ColE10	*mcr-4*	*bla*_TEM-1B_, *aph(4)-1a, aph(3’)-1c, aac(3)-IVa, aac(3)-IIa, strA, aadA1, floR, catA1, sul2, sul1, tet(B), dfrA1, mph(A), erm(B), mphB*	8
**4287C (conjugant in CSH26 Rif^R^) **			ColE10, I2	*mcr-4*		4
**CSH26 Rif^R^**						0.25
**R4280** ***E. coli***	Belgium	10 (CC10)	I1, FII, FIB, FIC, HI2, Col(MG828), ColRNAI_34, ColE10	*mcr-4*	*bla*_TEM-1B_, *aph4–1a, aac(3)-IVa, strB, aadA1, floR, catA1, sul1, tet(B), dfrA1, mph(B)*	8
**R4278** ***E. coli***	Belgium	7029	I1, X1, FII(pCoo), ColE10	*mcr-4*	*bla*_TEM-1B_, *strA, strB, aadA1, sul2, sul1, tet(A), dfrA1*	16

MIC: minimum inhibitory concentration; MLST: multilocus sequence typing.

**Figure 1:**
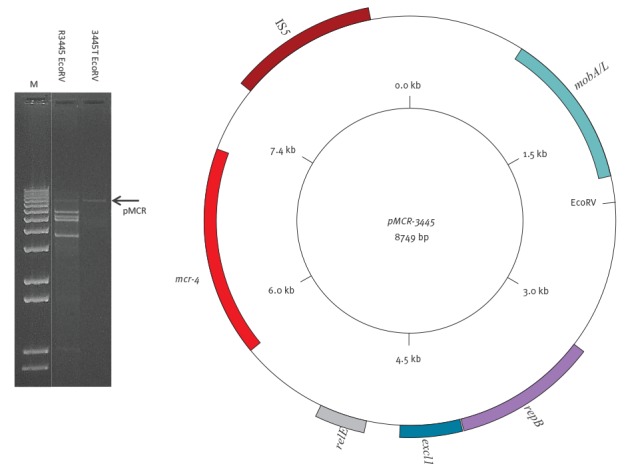
Plasmid content of the *Salmonella* isolate from swine, Italy 2013 (n = 1) and its transformant 3445T and map of the pMCR plasmid carrying the *mcr-4* gene

The 8,749 bp ColE10 plasmid (named pMCR; Figure 1) encoded the RepB replicase, MobA/L mobilisation proteins and the toxin RelE, and showed 99% nucleotide identity, 65% coverage, with the pPSP_ee2 *Pantoea* spp. plasmid (CP009884).In pMCR an IS*5* element (IS*Kpn6*) flanked a region showing 99% nucleotide identity with the genome of *Sh. frigidimarina* (CP000447.1). The assembly of pMCR was confirmed by PCR-based closure, using the primers listed in Table 2.

Left panel: M: 1 Kb Plus DNA ladder (Invitrogen, Italy). *Eco*RV-restricted plasmids purified from the R3445 *Salmonella* strain and the pMCR plasmid purified from the 3445T strain obtained by transformation (indicated by the arrow). Among the five ColE-like plasmids predicted by WGS, the pMCR ColE10 was estimated to be 8.7 kb, three copies per cell, while the other four ColE1 ranged from 7.0 to 4.0 kb in size and were estimated at more than 10 copies per cell.

Right panel: map of pMCR in the R3445 genome (http://wolfe.ucd.ie/GenomeVx/). The sequence of this plasmid is released at the NCBI GenBank under accession number MF543359.

**Table 2:** Primers used in this study for the detection of the new *mcr-4* gene

Primer name	Sequence	Target	Amplicon size
Mcr-4 FW	ATTGGGATAGTCGCCTTTTT	primers for the screening of the new *mcr-4 gene*	487 bp
Mcr-4 RV	TTACAGCCAGAATCATTATCA		
Mcr-4 ext FW	ATCTGTTAAGTTTGTTGGTGAC	external primers to amplify the complete *mcr-4* gene	1,820 bp
Mcr-4 ext RV	TGAGAGCTAAATGTAACAATAGA		
ColE10_repBF	TAAAGCATCGTGTAGTGTTTT	primers for the detection of the *repB* of the ColE10 plasmid	420 bp
ColE10_repBR	ATATAATCTGGAACATGTTAAG		
IS5FW	CATGACCTCAATCAGCTGG	In combination with ColE10_repBF, used for PCR-based closure of pMCR	4,588 bp
IS5 RV	TTTACTGAGATCTCTCCCAC	In combination with Mcr-4-RV, used for PCR-based closure of pMCR	2,143 bp

**Figure 2:**
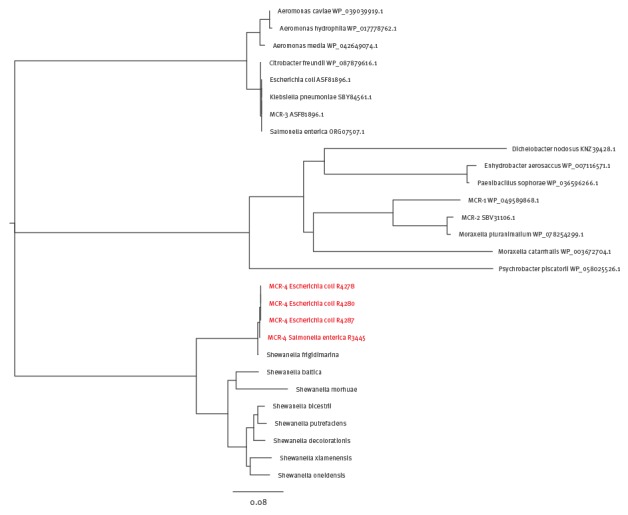
**Phylogenetic analysis of the entire MCR-4 protein sequence**

The *Sh. frigidimarina* region in pMCR encoded only one large protein that had an amino acid sequence with 82.0%–99.0% identity to phosphoethanolamine transferases found in *Shewanella* species and 34.0%, 35.0% and 49.0% amino acid sequence identity to MCR-1, MCR-2 and MCR-3 respectively (Figure 2).

Phylogenetic tree of the deduced amino acid sequences of putative phosphoethanolamine transferases from different bacterial species, in relation to the MCR-4-positive *Salmonella* isolate from swine, Italy 2013 and *E. coli* isolated in Europe in 2016, generated by bootstrap analysis using 1,000 replicates. The analysis was carried out using Geneious, version 10.1.3 [10]. Isolates in this study are highlighted in red.

Conjugation experiments failed to transfer the pMCR plasmid into an *E. coli* laboratory recipient, but purified plasmid DNA obtained from the R3445 *Salmonella* strain successfully transformed chemically competent DH5-alpha *E. coli* cells (Invitrogen, Italy), selecting the transformation on Luria-Bertani agar solid media plates containing 1 mg/L of colistin sulfate. The pMCR transformants showed a MIC of 2 mg/L for colistin, eightfold higher than the empty DH5-alpha recipient (MIC = 0.25 mg/L). pMCR plasmids in the transformants were analysed by restriction and PCR and found identical to that detected in the *Salmonella* strain (primers in Table 2). The phosphoethanolamine transferase identified on pMCR has been named MCR-4 (GenBank accession number: MF543359).

## The novel *mcr*-*4* gene in *Escherichia coli* strains from pigs in Europe

A total of 125 *E. coli* isolates were collected in Italy (34 strains), Spain (43 strains) and Belgium (48 strains) in 2015 and 2016, from specimens submitted for routine diagnosis of post-weaning diarrhoea in piglets. Fifty isolates were colistin-resistant with MIC > 2 mg/L [3]. A PCR screening for *mcr* was performed on the entire collection of 125 isolates: 32 strains were positive for *mcr-1* [6], three for *mcr-2* [7] and 11 for the novel *mcr4* gene identified in the *Salmonella* isolate R3445 (primers used are shown in Table 2). Nine strains were from piglets from Spain and two from Belgium. The eleven *mcr-4-*positive strains were also positive for the ColE10 replicase and showed a MIC ≥ 4 mg/L for colistin.

WGSs of three *E. coli* positive for the new *mcr-4* gene, were obtained (R4278, R4280 and R4287 in Table 1). The pMCR plasmid was detected in the three genomes, but several additional plasmids were also identified, belonging to the I1, I2, F, X1 and HI2 families, probably associated with the large repertoire of resistance genes in these *E. coli* strains (Table 1).

The pMCR plasmid was transferred by conjugation at 37 °C, at a 1 × 10^−4^ frequency (conjugants/donors colony-forming units), from strain R4287 to a rifampicin-resistant CSH26 *E. coli* recipient strain. Conjugants were screened by plating 10-fold serial dilutions of the mating mixture on Luria-Bertani agar solid media plates containing 2 mg/L of colistin sulfate and 100 mg/L rifampicin, as resistance markers for pMCR and CSH26, respectively.

All transconjugants contained the pMCR and a co-resident I2 plasmid that acted as the helper plasmid promoting conjugation. The R4287 pMCR conjugants showed MIC = 4 mg/L for colistin, 16-fold higher than the empty CSH26 recipient (Table 1). From strain R4280, pMCR was not transferred by conjugation. Interestingly, this strain did not carry the I2 helper plasmid that was present in R4287.

In the R4278 genome, the pMCR was located on the chromosome integrated within a Type I methylation gene. Two IS5 copies in direct orientation flanked the complete pMCR plasmid sequence, suggesting that a transposition event has driven the chromosomal integration of the plasmid.

## Conclusions

The chromosomal mechanism of colistin resistance in *Enterobacteriaceae* involves mutations in several genes involved in decoration and structural modifications of lipopolysaccharides (LPS) [8]. Plasmid-borne *mcr* genes encoding phosphoethanolamine transferases, modifying the lipid A have been described as an important mechanism conferring decreased susceptibility to colistin. Since its discovery, the *mcr-1* gene has been widely detected in enterobacteria isolated from animals and humans in all the world [8]. In contrast, the *mcr-2* variant has been rarely identified, and only in *E. coli* strains of swine and bovine origin in Belgium [7]. The recently discovered *mcr-3* gene has been identified in *E. coli*, *Klebsiella pneumoniae* and *Salmonella* from Asia and the United States [9].

Our findings demonstrate the presence of a new plasmid-mediated colistin *mcr-4* gene on a small, not self-conjugative plasmid. ColE are plasmids with a broad host range that can be expected to be able to replicate in different bacterial species and genera. However, the mobilisation of the plasmid needs a helper plasmid to promote conjugation.

We found the *mcr-4* gene in a monophasic variant of *Salmonella* Typhimurium isolated from a pig at slaughter in Italy and in 11 of 125 (9%) *E. coli* isolates from piglets. Nine of 43 *E. coli* from Spanish piglets were positive, 37% (9/24) of strains from Spain with a colistin MIC > 2 mg/L. Among the Belgian strains, two positive *E. coli* among 15 colistin-resistant isolates were found. These findings suggest considerable dissemination of the novel gene in Europe. Screening for this *mcr-4* variant should be immediately implemented in zoonotic bacteria, including *Salmonella* and *E. coli* from food-producing animals and human sources to understand its diffusion in Europe, and to evaluate the risk for human health posed by this novel plasmid-mediated colistin resistance determinant.
